# Women's Health Care Utilization among Harder-to-Reach HIV-Infected Women ever on Antiretroviral Therapy in British Columbia

**DOI:** 10.1155/2012/560361

**Published:** 2012-11-26

**Authors:** Xuetao Wang, Kate A. Salters, Wen Zhang, Lawrence McCandless, Deborah Money, Neora Pick, Julio S. G. Montaner, Robert S. Hogg, Angela Kaida

**Affiliations:** ^1^Faculty of Health Sciences, Simon Fraser University, 8888 University Drive, Burnaby, BC, Canada V5A 1S6; ^2^BC Center for Excellence in HIV/AIDS, 608-1081 Burrard Street, Vancouver, BC, Canada V6Z 1Y6; ^3^Department of Obstetrics and Gynecology, Faculty of Medicine, University of British Columbia, Vancouver, BC, Canada V6T 1Z3; ^4^BC Women's Hospital and Health Centre, 4500 Oak Street, Vancouver, BC, Canada V6H 3N1; ^5^Department of Medicine, Faculty of Medicine, University of British Columbia, Vancouver, BC, Canada V6T 1Z3

## Abstract

*Background*. HIV-infected women are disproportionately burdened by gynaecological complications, psychological disorders, and certain sexually transmitted infections that may not be adequately addressed by HIV-specific care. We estimate the prevalence and covariates of women's health care (WHC) utilization among harder-to-reach, treatment-experienced HIV-infected women in British Columbia (BC), Canada. *Methods*. We used survey data from 231 HIV-infected, treatment-experienced women enrolled in the Longitudinal Investigations into Supportive and Ancillary Health Services (LISA) study, which recruited harder-to-reach populations, including aboriginal people and individuals using injection drugs. Independent covariates of interest included sociodemographic, psychosocial, behavioural, individual health status, structural factors, and HIV clinical variables. Logistic regression was used to generate adjusted estimates of associations between use of WHC and covariates of interest. *Results*. Overall, 77% of women reported regularly utilizing WHC. WHC utilization varied significantly by region of residence (*P* value <0.01). In addition, women with lower annual income (AOR (95% CI) = 0.14 (0.04–0.54)), who used illicit drugs (AOR (95% CI) = 0.42 (0.19–0.92)) and who had lower provider trust (AOR (95% CI) = 0.97 (0.95–0.99)), were significantly less likely to report using WHC. *Conclusion*. A health service gap exists along geographical and social axes for harder-to-reach HIV-infected women in BC. Women-centered WHC and HIV-specific care should be streamlined and integrated to better address women's holistic health.

## 1. Introduction

More than 30 years into the global HIV/AIDS epidemic, 33.3 million people worldwide are currently living with HIV, of whom more than 50% are women and girls [1]. In Canada, the proportion of women among newly reported HIV cases has increased from less than 5% in 1985 to 26% in 2009 [2]. In the same year, the number of women living with HIV in Canada was estimated to be 12,000, of whom approximately 2860 resided in the province of British Columbia (BC) [2]. The proportion of HIV in younger age groups is also higher for women than for men [2, 3], suggesting that women are contracting HIV at a younger age than men. The growing feminization of the HIV epidemic means that more young women of reproductive age will be living with HIV in Canada. 

HIV infection is associated with many women's health issues that may not be adequately addressed in HIV-specific care [4, 5]. HIV-infected women are more likely than their HIV-negative counterparts to have abnormal gynaecological conditions, such as squamous intraepithelial lesions, cervical cancer, and both bacterial and viral sexually transmitted infections [6–11]. Psychological disorders such as depression are also more prevalent among HIV-infected women than HIV-negative women, likely due to HIV-related stigma and lack of social support [12, 13]. In addition, HIV-infected women's health focus changes as they go through different stages of life. Women of younger age may focus on menstrual cycle disorders and family planning, while women aging with HIV might need support managing menopause symptoms and their heightened risk of osteoporosis [6]. The introduction of highly active antiretroviral therapy (HAART) has markedly prolonged the life expectancy and greatly increased the reproductive expectations of HIV-infected women [6], but HAART alone cannot address the multiple realms of health impacted by one's HIV status. 

Women's health care (WHC), including regular gynaecological care and counselling, has been shown to improve women's multiple health outcomes and should be provided along with HIV-specific care to ensure a more integrated and holistic care model centered on patients' need [14–16]. The World Health Organization (WHO) has proposed a framework to strengthen the linkage between WHC and HIV care at both policy and program levels [17]. However, studies have shown an underutilization of women's health and gynaecological services among HIV-infected women in the US and eastern Canada [18–20].

This study investigates the uptake of WHC among a sample of harder-to-reach and treatment-experienced HIV-infected women in BC, Canada and the factors associated with utilization. Studies on the intersection between HIV and WHC may provide suggestions for mitigating the wide range of women's health issues associated with HIV infection by generating relevant program and policy recommendations.

## 2. Methods

### 2.1. Participants and Recruitment

We conducted our analyses using data from the Longitudinal Investigations into Supportive and Ancillary Health Services (LISA) study which was specifically designed to recruit harder-to-reach populations living with HIV who have ever accessed antiretroviral therapy (ART) in BC.

In BC, ART is distributed free of charge to all eligible people living with HIV/AIDS through the Drug Treatment Program (DTP) at the British Columbia Centre for Excellence in HIV/AIDS (BC-CfE) [21]. Medications are distributed in accordance with the guidelines set by the BC Therapeutic Guideline Committee, which have remained consistent with those from the International AIDS Society, USA between 1996 and at last revision in 2010 [22, 23]. Individuals are enrolled in DTP when they are first prescribed ART by their physicians. The physician must complete a drug request form detailing baseline information, such as CD4 cell counts, plasma HIV RNA levels, and past HIV-specific drug history [21]. Patients on ART are then typically monitored by the prescribing physicians at intervals no longer than three months [24]. As of June 2012, approximately 5500 patients are currently enrolled in the DTP [25]. 

In order to evaluate the impact of the social determinants of health on the health care utilization and clinical outcomes of harder-to-reach HIV-infected individuals who have accessed ART in BC, the LISA study was established. The LISA study consists of a comprehensive cross-sectional survey, which collected data on participants' sociodemographic information, service utilization, and quality of life. Individuals who have ever enrolled in the DTP (or who had initiated ART any time prior to the interview) and who were at least 19 years of age at the time of interview were eligible to participate in the LISA study. Between July 2007 and January 2010, all 9514 eligible participants enrolled in the DTP were targeted through study information letters distributed by physicians providing HIV care and at pharmacies when patients refilled ART prescriptions. In addition to these targeted recruitment strategies, participants were recruited to participate in LISA through notices posted at HIV/AIDS clinics and service organizations across the province and via word-of-mouth advertising. A result of this convenience sampling was an overrepresentation of individuals who were already accessing HIV-related services. The study sought to enroll 1000 individuals and to oversample particular subpopulations (including women, people who inject drugs, and people who identified themselves as aboriginal) to provide sufficient power for subanalyses. Therefore, the LISA cohort enrolled a nonprobability sample of DTP patients and represented more marginalized populations with some level of access to HIV-related services. 

The LISA questionnaire, which was developed, reviewed, and piloted with a team of researchers and a community advisory board, was administered throughout BC by trained interviewers at various clinics, HIV/AIDS service organizations, or by telephone. The questionnaire took approximately 60 minutes to complete, and a $20 honorarium was offered to each participant. The financial incentive resulted in an oversampling of harder-to-reach populations.

For this analysis, inclusion was restricted to LISA participants who identified themselves as females (versus male or transgender) and who had HIV clinical data available through the DTP.

Ethical approval for the LISA study was obtained from the University of British Columbia/Providence Health Care, Simon Fraser University, the University of Victoria, and the Vancouver Coastal Health Research Ethics Boards. 

### 2.2. Measures

#### 2.2.1. Outcome Variable

The primary outcome variable “WHC utilization” was assessed via self-report based on responses to the statement “*I have a physician who I see regularly for women's health care [e.g., pap smear, gynaecology].*” Responses were dichotomized into “Yes, utilizes WHC” (based on those who responded “definitely true” or “somewhat true”) or “No, does not utilize WHC” (based on those who responded “definitely false” or “somewhat false”). A small number of women who responded “neither true nor false” (*n* = 5) or who did not respond (*n* = 15) were excluded from this analysis. 

#### 2.2.2. Explanatory Variables of Interest

The demographic variables included age at interview, aboriginal ancestry (yes versus no), health authority (HA) (based on patient address), rural residency (rural versus urban), and marital status (yes versus no; yes defined as being legally married or common-law). 

 Sociodemographic variables included education (high school or greater versus others), current employment (yes versus no), personal annual income (<$15,000 versus ≥$15,000; including wages, salaries, net self-employment, and any other income such as welfare), housing stability (yes versus no; stable housing defined as living in a house or apartment), and food security (yes versus no). Food security was measured based on a modified version of the Radimer/Cornell Questionnaire, and food insecurity was defined as having at least one positive answer to any of the 13 questions [26, 27].

Psycho-social variables included HIV-related stigma, perceived neighbourhood problems, perceived neighbourhood cohesion, and quality of life (QoL). HIV-related stigma was measured using the validated HIV stigma scale ranging from 0 to 100, with higher values corresponding to higher HIV-related stigma [28]. Perceived neighbourhood problems and perceived neighbourhood cohesion were assessed using the scales developed by Ellaway et al., and the original scales were transformed to range from 0 to 100, with higher values indicating higher perceptions of more neighbourhood problems or more neighbourhood cohesion [29]. The QoL of participants was assessed using the validated HIV/AIDS-targeted quality of life (HAT-QoL) instrument [30]. The HAT-QoL scale has 9 dimensions: overall function, sexual function, disclosure worries, health worries, financial worries, HIV mastery, life satisfaction, medical worries, and provider trust. Each measure has a scale from 0 to 100, with higher scores corresponding to better QoL. 

Behavioural variables included alcohol use at time of interview, illicit drug use at time of interview (illicit drugs including cocaine, crack, heroin, speedball, crystal meth, steroids, ecstasy, hallucinogens, GHB, and special K), injection of drugs at time of interview (drugs for injection including cocaine, crack, heroin, speedball, and crystal meth), sexual activity (active versus inactive; active defined as having >0 sexual activities in last 6 months), condom use of sexually active participants (yes versus no; yes being defined as using condom 100% of the time during vaginal sex in the last 6 months), sex trade history (yes versus no), pregnancy intention (yes versus no), and number of births in life time. 

Individual health status variables included history of sexually transmitted infections (HPV, Chlamydia, gonorrhoea, syphilis), abnormal Pap smear in the last 6 months (yes versus no), and symptoms of depression (yes versus no). The validated 10-item Center for Epidemiological Studies-Depression (CES-D10) scale was used to assess symptoms of depression, with scores of 10 or higher being defined as having symptoms of depression [31].

HIV clinical variables were obtained from DTP and included ART status at time of interview (yes versus no), CD4 count at time of interview (obtained from the most recent test result prior to interview), plasma viral load (pVL, log 10/mL) at time of interview (obtained from the most recent test result prior to interview), and VL suppression (defined as having two consecutive tests of VL < 250 copies/mL 12 months prior to interview).

### 2.3. Statistical Analyses

The prevalence of WHC utilization among LISA participants was estimated. Bivariate analyses were then performed to examine associations between each explanatory variable of interest and utilization of WHC. Chi-squared tests were used to compare categorical variables; Wilcoxon's rank sum test was used to compare continuous variables. Marginally associated variables (*P* < 0.2) based on the bivariate analyses were included in an exploratory multivariable logistic regression model to identify independent factors associated with WHC utilization. A backward selection procedure using the Akaike Information Criterion (AIC) was performed to select variables (*P* < 0.05) to be included in the final model [32]. All analyses were conducted using SAS 9.2 (SAS Inc., Cary, NC).

## 3. Results

**Figure 1:**
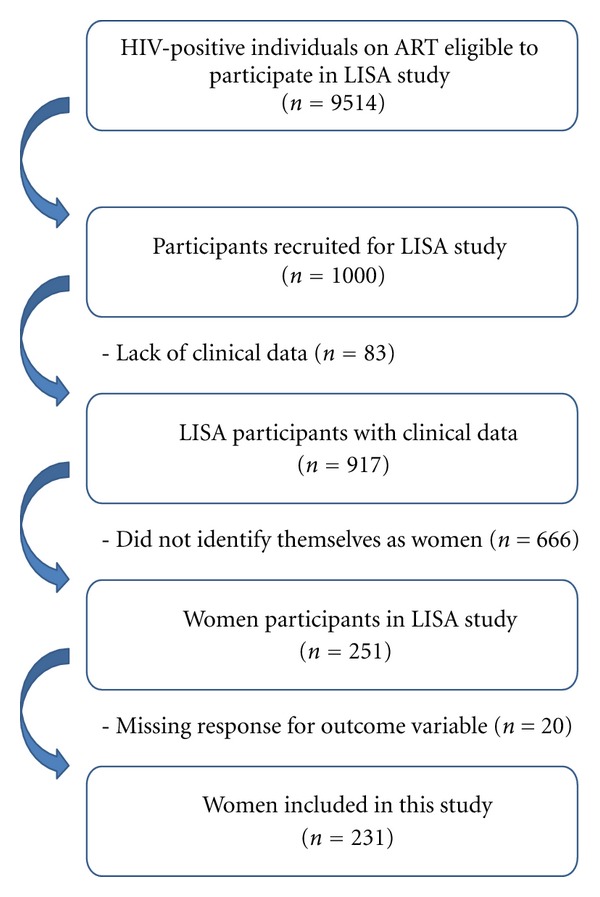
Selection of study subjects from the original LISA cohort.

A total of 1000 participants were recruited and interviewed for the LISA study from July 2007 to January 2010; however, only 917 were successfully linked to clinical data in the DTP, as shown in Figure 1. Out of the 917 participants with clinical data available, there were 231 women who were included in this analysis. Overall, 179 (77%) of the HIV-infected, treatment-experienced women reported utilizing WHC. 

Table 1 describes the baseline characteristics of the women included in this study. The median age was 41 years (interquartile range (IQR): 34–46). Half (49%) of the participants reported being of aboriginal ancestry. The geographical distribution of the participants was 60% in Vancouver coastal HA, the more populated Pacific coast of southwestern BC, 5% in Interior HA, BC's southern interior region including many rural communities, 4% in Northern HA, northern BC consisting of a multitude of rural and First Nations communities, 10% in Vancouver island HA, mainly the islands to the west of Vancouver, and 21% in Fraser HA, a populated region to the east of Vancouver. About half (47%) of the participants had an education level of high school or greater, 15% were currently employed and 72% reported an annual income below $15,000. Housing was stable for 62% of the women and 23% were food secure. 

Table 1 also compares the characteristics of women who utilized WHC and those who did not. The geographical distribution was significantly different (*P* < 0.01), with a higher proportion living in Vancouver coastal HA (63% versus 49%) and a lower proportion in Vancouver island HA (6% versus 24%) for women who utilized WHC compared with women who did not. Women who utilized WHC were less likely to use illicit drugs (*P* < 0.01), more likely to be employed (*P* = 0.04), less likely to have an annual income <$15,000 (*P* < 0.01), more likely to have stable housing (*P* = 0.02), and more likely to have a higher score for perceived neighbourhood cohesion (*P* = 0.02) and provider trust (*P* < 0.01). Although women who utilized WHC had higher percentage of ART use at the time of interview and higher rate of VL suppression, none of the HIV-related clinical variables showed significant differences between the two comparison groups. 

Table 2 displays the results of the multivariable logistic regression model and shows that the adjusted odds ratios were consistent with the unadjusted odds ratios. The adjusted odds of WHC utilization among participants were 88% lower in Vancouver island HA compared with Vancouver coastal HA (AOR = 0.12, 95 CI (0.04–0.37)), 58% lower among illicit drug users compared with nondrug users (AOR = 0.42, 95 CI (0.19, 0.92)), and 86% lower for those with income <$15,000 compared with those with income ≥$15,000 (AOR = 0.14, 95 CI (0.04, 0.54)). A one-unit decrease in the provider trust scale was associated with a 3% decrease in the adjusted odds of WHC utilization (AOR = 0.97, 95 CI (0.95, 0.99)). 

## 4. Discussion

Our results demonstrate the existence of a health service gap along geographical and social axes for harder-to-reach HIV-infected women who have accessed ART in BC. We found that 77% of the women in the LISA cohort used WHC. This study is among the first to assess the prevalence of WHC utilization among HIV-infected women as the previous literature documented utilization of gynaecological service only. 

This study shows that poorer socioeconomic status is associated with women's ability to access WHC, despite a context that theoretically provides universal health care. Income-related inequalities in health care utilization have been well documented in countries with universal health care systems [33]. In Canada, lower income has been shown to be associated with less contact with general practitioners and specialists and decreased use of surgical day care [34, 35]. Several qualitative studies have examined the barriers to health care experienced by low-income Canadians and found some of the major barriers to be transportation cost, lack of childcare, and a lack of integration of health services [36, 37]. The socioeconomic inequality in WHC utilization demonstrated in this study points to the necessity of providing health services tailored to the needs of low-income women, which may include outreach and providing transportation reimbursement, food, and free childcare during clinic visits. Furthermore, while struggling to meet their basic needs, low-income women might have other competing demands in life such as food and housing. The fact that stable housing and employment were marginally associated with WHC utilization in this study speaks to the need for socio-economic policy reform in BC that addresses structural determinants of health such as housing affordability and unemployment. 

Utilization of WHC was negatively associated with illicit drug use. This is consistent with findings from previous studies investigating the relationship between illicit drug use and gynaecological service utilization [38, 39]. It is worth noting that drug injection was not associated with the outcome in this study. This may indicate that the method of drug use does not innately influence access to WHC, but rather, that stigma and social barriers exist for all illicit drug users [37, 40, 41]. The exact pathways linking illicit drug use and WHC utilization are not clear and require further qualitative studies. 

Lower provider trust was associated with a lack of WHC utilization in this study. Trust in physicians has long been identified as a determining factor for patient's utilization of health care services [42, 43], but this study has revealed a more specific relationship, namely, between trust in the physician who provides regular HIV care and women's utilization of WHC. This indicates the pivotal role of HIV care physicians in influencing HIV-infected women's health seeking behaviour. This cohort had a high score of provider trust, with a median of 92 for women who utilized WHC and 83 for women who did not. Therefore, women in the cohort were generally satisfied with the HIV care they received. In order to further improve physician trust, evidence suggests that women should be provided with a safe, nonjudgemental clinical environment and an option of seeing a female care provider who could improve patient-physician relationship by making women feel safer and more understood, especially when gynaecological care is involved [37]. 

The geographical variation in WHC utilization may reflect the inequity of health service provision in rural versus urban areas of BC, where larger communities in Vancouver coastal HA generally have more comprehensive and easily accessible health services than smaller communities in Vancouver island HA [44]. However, due to the large proportion of missing data for participants' rural residency status, the exact reasons behind this geographical variation could not be determined, and further studies are warranted. 

HIV-related clinical variables did not vary by WHC utilization status, suggesting that all women in this study responded similarly to HIV-specific treatment. The lack of association between WHC and HIV clinical variables reflects a lack of integration between WHC and HIV care and a deficiency in women's holistic care in BC. Centralizing and streamlining WHC with HIV-specific care should be emphasized to avoid unnecessary shuffling of patients within the health care system. WHC could be tailored to low-income women's financial need by providing transportation reimbursement and free childcare and could increase patient's provider trust by offering a safe, nonjudgemental multidisciplinary environment and the option of seeing a female care provider. These suggestions echo the best practices in promoting HIV-infected women's health [45–47]. Oak Tree Clinic (OTC) located in Vancouver has been a pioneer in implementing women-centered care for HIV-infected women in BC. The clinic is a provincial resource for HIV-infected women and children, with HIV care, obstetrical and gynaecological care, dietary care, and social support all amalgamated within one facility [48]. The fact that a quarter of the women in this study were recruited from the OTC could explain the relatively high prevalence of WHC utilization among study participants. 

An ad hoc analysis was done comparing WHC utilization between women who accessed care at OTC and women who did not. As shown in Supplemental Table 1 available online at doi:10.1155/2012/560361, WHC utilization and access to OTC was highly associated (*P* < 0.01), and women who accessed OTC were significantly more likely to report WHC utilization than women who did not access OTC. This may be related to the fact that health care providers at the OTC are highly sensitized to the importance of WHC and incorporate WHC to routine HIV care. The outreach social workers and nurses, as well as free food and child care at the OTC, also help bring in harder-to-reach women, such as women who inject drugs and who have low income. Furthermore, OTC provides a safe women's majority (75%) environment to help build trust, and the clinicians have developed long-term relationship with the patients. The multidisciplinary team works to increase women's engagement and ability to deal with the HIV challenges. This ad hoc analysis provided strong evidence that women-centered multidisciplinary service integration could effectively encourage WHC utilization and promote health among HIV-infected women, especially the harder-to-reach women. As the next step, studies could be designed to more thoroughly evaluate OTC's model of care.

Readers should be cautious when interpreting our findings to the general population of women infected with HIV in the province. Oversampling of injection drug users and aboriginal people means that women in this cohort may encounter more social vulnerability. The financial incentives provided to all LISA study participants might have introduced volunteer bias, where women in need of financial gain, particularly those who reside in the impoverished downtown east side of Vancouver, were overrepresented. As LISA study participants have accessed HIV treatment and were recruited through physicians, clinics, and AIDS service organizations, those interviewed were already connected to care in some degree. Therefore, our sample does not include those individuals who are not able to access care and may face several structural barriers to care and thus may not represent the most marginalized people living with HIV in BC. Second, the variables used in this study were self-reported and, therefore, subject to recall bias and social desirability bias. Future studies could use physician or hospital records to objectively assess WHC utilization among HIV-infected women who have accessed ART. Third, the definition of WHC might vary among study participants. Some might interpret it as services including a Pap smear, while others might consider it a full range of services tailored towards women. Future studies could divide this broader measure of WHC into more specific type of services. Fourth, automated backward selection procedure ran the risk of eliminating variables that are meaningfully associated with the outcome solely based on statistical significance, but it provided an objective way of model selection. Lastly, the cross-sectional design of this study means that causality cannot be inferred in the relationship between WHC utilization and the covariates. However, previous qualitative studies have revealed some of the social pathways linking WHC utilization with income and provider trust, rendering policy recommendations more relevant and persuasive. 

In conclusion, our findings suggest that there is a health service gap for harder-to-reach HIV-infected women who have accessed ART in BC. Improvements could be made by enhancing the integration between HIV-specific care and WHC and providing women-centered health services. Socio-economic policy reforms are also needed to address the structural inequalities in health. 

## Supplementary Material

The table shows that out of the 56 women who had access to Oak Tree Clinic (OTC), 53 responded “Yes” to WHC utilization and only 3 answered “No”. The significant association (*P* < 0.01) between WHC utilization and access to OTC suggests that women who accessed OTC were significantly more likely to report WHC utilization than women who did not access OTC. This ad hoc analysis provided strong evidence that women-centered multidisciplinary service integration, pioneered by OTC, could effectively encourage WHC utilization and promote health among harder-to-reach HIV-infected women.

## Figures and Tables

**Table 1:** Baseline characteristics of the HIV-infected women in the LISA cohort (*n* = 231) by women's health care (WHC) utilization (yes versus no).

	WHC utilization			*P* value
	All (*n* = 231)	Yes (*n* = 179)	No (*n* = 52)
	Demographic variables			

Age at interview (years) (median (IQR))	41 (34–46)	41 (34–46)	41 (36–46)	0.70
Aboriginal ancestry (% Y)	114 (49%)	87 (49%)	27 (52%)	0.67
Health authority (HA)				<0.01
(i) Vancouver coastal HA	136 (60%)	111 (63%)	25 (49%)	
(ii) Interior HA	11 (5%)	10 (6%)	1 (2%)	
(iii) Northern HA	10 (4%)	7 (4%)	3 (6%)	
(iv) Vancouver island HA	22 (10%)	10 (6%)	12 (24%)	
(v) Fraser HA	48 (21%)	38 (22%)	10 (20%)	
Rural residency (% rural)*	3 (2%)	3 (2%)	0 (0%)	0.99
Marital status (% Y)	42 (18%)	29 (16%)	13 (25%)	0.15

	Sociodemographic variables			

High school or greater (% Y)	108 (47%)	87 (49%)	21 (40%)	0.30
Employment (% Y)	34 (15%)	31 (17%)	3 (6%)	0.04
Annual income < $15,000 (% Y)	166 (72%)	119 (66%)	47 (92%)	<0.01
Housing stability (% Y)	143 (62%)	118 (66%)	25 (48%)	0.02
Food security (% Y)	54 (23%)	44 (25%)	10 (19%)	0.41

	Psychosocial variables			

HIV-related stigma (scale, median (IQR))	53 (38–65)	53 (38–68)	50 (38–65)	0.63
Perceived neighbourhood problems (scale, median (IQR))	35 (15–62)	35 (15–62)	35 (19–62)	0.98
Perceived neighbourhood cohesion (scale, median (IQR))	56 (43–66)	56 (44–69)	51 (35–62)	0.02
Quality of life (scale, median (IQR))				
(i) Overall function	43 (29–64)	43 (25–64)	43 (32–64)	0.88
(ii) Life satisfaction	69 (50–75)	69 (53–78)	63 (47–75)	0.07
(iii) Health worry	45 (30–65)	45 (30–65)	45 (25–65)	0.79
(iv) Financial worry	38 (19–63)	38 (19–63)	38 (25–56)	0.50
(v) Medical worry	69 (56–75)	69 (56–75)	63 (56–75)	0.10
(vi) Disclosure worry	50 (35–65)	50 (35–65)	55 (35–65)	0.67
(vii) HIV Mastery	50 (42–75)	58 (42–75)	50 (42–75)	0.79
(viii) Provider trust	92 (75–100)	92 (75–100)	83 (75–100)	<0.01
(ix) Sexual function	50 (42–67)	50 (42–67)	50 (42–58)	0.58

	Behavioural variables			

Alcohol use at time of interview (% Y)	105 (48%)	79 (47%)	26 (52%)	0.54
Illicit drug use at time of interview (% Y)	123 (53%)	85 (48%)	38 (73%)	<0.01
Injection of drugs at time of interview (% Y)	53 (23%)	37 (21%)	16 (31%)	0.13
Sexually active in last 6 months (% Y)	125 (54%)	100 (56%)	25 (48%)	0.32
Condom use of sexually active participants** (% Y)	46 (40%)	36 (40%)	10 (42%)	0.85
Sex trade history*** (% Y)	71 (57%)	52 (55%)	19 (66%)	0.30
Pregnancy intention (% Y)	32 (14%)	26 (15%)	6 (12%)	0.58
Number of births in life time (median (IQR))	2 (1–3)	2 (1–4)	2 (1–3)	0.43

	Individual health status variables			

Ever diagnosed with HPV (% Y)	18 (8%)	15 (8%)	3 (6%)	0.53
Ever diagnosed with chlamydia (% Y)	48 (21%)	33 (18%)	15 (29%)	0.10
Ever diagnosed with gonorrhea (% Y)	38 (16%)	28 (16%)	10 (19%)	0.54
Ever diagnosed with syphilis (% Y)	22 (10%)	15 (8%)	7 (13%)	0.27
Abnormal Pap smear in last 6 months (% Y)****	34 (17%)	28 (17%)	6 (15%)	0.75
Symptoms of depression (% Y)	156 (68%)	121 (68%)	35 (67%)	0.97

	HIV clinical variables			

ART status at time of interview (% Y)	177 (77%)	141 (79%)	36 (69%)	0.15
CD4 count at time of interview (median (IQR))	300 (170–470)	290 (170–470)	310 (175–475)	0.85
pVL (log 10) at time of interview (median (IQR))	1.7 (1.5–3.1)	1.7 (1.5–3.1)	1.7 (1.5–3.0)	0.46
VL suppression (% Y)	140 (61%)	111 (62%)	29 (56%)	0.42

*30% of the data are missing (*n* = 162).

**50% of the data are missing (*n* = 116).

***46% of the data are missing (*n* = 125).

****only participants who answered “yes” or “no” were included in the denominator.

**Table 2:** Multivariate analysis of factors associated with women's health care (WHC) utilization among HIV-infected women in the LISA cohort.

	Unadjusted		Adjusted	
Variable	odds ratio (OR)	*P* value	odds ratio (AOR)	*P* value
	(95% CI)		(95% CI)	
Health authority (HA)		<0.01		<0.01
Vancouver coastal HA	1.00 (—)		1.00 (—)	
Fraser HA	0.86 (0.38–1.95)		0.52 (0.21–1.29)	
Interior HA	2.25 (0.28–18.40)		1.78 (0.19–16.79)	
Northern HA	0.53 (0.13–2.18)		0.43 (0.09–2.07)	
Vancouver island HA	0.19 (0.07–0.48)		0.12 (0.04–0.37)	
Annual income		<0.01		<0.01
≥C$15,000	1.00 (—)		1.00 (—)	
<C$15,000	0.17 (0.06–0.49)		0.14 (0.04–0.54)	
Illicit drug use		<0.01		0.03
No	1.00 (—)		1.00 (—)	
Yes	0.34 (0.17–0.66)		0.42 (0.19–0.92)	
Provider trust (QoL scale)	0.96 (0.94–0.98)	0.03	0.97 (0.95–0.99)	0.03
