# Supplementary material for: Women's Health Care Utilization among Harder-to-Reach HIV-Infected Women ever on Antiretroviral Therapy in British Columbia
Source: AIDS Res Treat. 2012 Nov 26;2012:560361. doi: 10.1155/2012/560361 (PMC3513717; doi:10.1155/2012/560361)
Supplement: Supplementary file 1 — The table shows that out of the 56 women who had access to Oak Tree Clinic (OTC), 53 responded “Yes” to WHC utilization and only 3 answered “No”. The significant association (P < 0.01) between WHC utilization and access to OTC suggests that women who accessed OTC were significantly more likely to report WHC utilization than women who did not access OTC. This ad hoc analysis provided strong evidence that women-centered multidisciplinary service integration, pioneered by OTC, could effectively encourage WHC utilization and promote health among harder-to-reach HIV-infected women. [file 560361.f1.docx]

**Supplemental Table 1. Ad-hoc analysis of the association between access to Oak Tree Clinic and Women’s Health Care (WHC) utilization among HIV-infected women in the LISA cohort.**

|  | **WHC utilization** | | |  |
| --- | --- | --- | --- | --- |
|  | **All (n=231)** | **Yes (n=179)** | **No (n=52)** | ***p*-value** |
| **Access to Oak Tree Clinic (%Y)** | 56(24%) | 53(29%) | 3(6%) | <0.01 |
